# Characteristics of hydraulic fracture surface based on 3D scanning technology

**DOI:** 10.1098/rsos.190702

**Published:** 2019-05-01

**Authors:** Fan Zhang, Geng Ma, Yunqi Tao, Xiao Liu, Yixin Liu, Rui Li

We were happy to publish our paper entitled ‘Characteristics of Hydraulic Fracture Surface Based on 3D Scanning Technology’ in the journal *Royal Society Open Science* (https://doi.org/10.1098/rsos.171845). However, after serious consideration, due to some technical reasons (there are some errors in the operation of 3D scanning device) and miscalculation, we have decided to retract this article from *Royal Society Open Science*, the indexing services (PubMed, PubMed Central and the Directory of Open Access Journals) and the indexing from Scopus, Web of Science and the Astrophysics Data System (ADS). The details about the ‘technical reasons and miscalculations’ are provided below.

In the published article, a 3D scanning device was applied to scan the surface of fractured rock specimens and extract the surface characteristics. The 3D scanning technology presented is an effective tool for the quantitative study of fracture characteristics in hydraulic fracturing experiments. Firstly, before writing this paper, the experimenters were not trained professionally. A test on hydraulically fractured sample was conducted to obtain the characteristics of hydraulic fracture surface by 3D scanning technology. Secondly, after serious consideration, the test results were affected by many factors, including resolution of CCD sensor, angle and distance between fractured sample and OKIO-B-200 scanner, dye penetrant inspection material upon fractured sample and other factors. Though the superficial characteristics of fractured sample were obtained by 3D scanning technology, the influencing factors were not explained clearly in the published paper, which directly affected the accuracy of shaded relief model, 3D scanning image, contour map, superficial area and volume of the fractured sample. Finally, different experimental conditions lead to different experimental results. The technical reasons and miscalculation greatly influenced the academic credibility of the published paper. The conclusions may have a bad effect on the journal and readers globally. Therefore, we have decided to retract this paper from *Royal Society Open Science*.

